# Effects of Umbilical Cord Management Strategies on Stem Cell Transfusion, Delivery Room Adaptation, and Cerebral Oxygenation in Term and Late Preterm Infants

**DOI:** 10.3389/fped.2022.838444

**Published:** 2022-04-04

**Authors:** Emel Okulu, Sule Haskologlu, Deniz Guloglu, Ezgi Kostekci, Omer Erdeve, Begum Atasay, Acar Koc, Feride Soylemez, Figen Dogu, Aydan Ikinciogullari, Saadet Arsan

**Affiliations:** ^1^Division of Neonatology, Department of Pediatrics, Ankara University Faculty of Medicine, Ankara, Turkey; ^2^Division of Pediatric Immunology and Allergy, Department of Pediatrics, Ankara University Faculty of Medicine, Ankara, Turkey; ^3^Department of Obstetrics and Gynecology, Ankara University Faculty of Medicine, Ankara, Turkey

**Keywords:** umbilical cord blood, clamping, placental transfusion, endothelial progenitor cells (EPCs), CD34+ hematopoietic stem cell

## Abstract

**Background:**

The umbilical cord blood contains a high concentration of stem cells. There is not any published study evaluating the amount of stem cells that have the potential to be transferred to the infant through placental transfusion methods as delayed cord clamping (DCC) and umbilical cord milking (UCM). The aim of this study is to measure the concentrations of endothelial progenitor cell (EPC) and CD34+ hematopoietic stem cell (HSC) in the placental residual blood volume (PRBV), and evaluate the delivery room adaptation and cerebral oxygenation of these infants.

**Methods:**

Infants with ≥36 gestational weeks were randomized to receive DCC (120 s), UCM, or immediate cord clamping (ICC). EPC and CD34+ HSC were measured by flow cytometry from the cord blood. PRBV was collected in the setup. The cord blood gas analysis and complete blood count were performed. The heart rate (HR), oxygen saturation (SpO2), and cerebral regional oxygen saturation (crSO2) were recorded.

**Results:**

A total of 103 infants were evaluated. The amount of PRBV (in ml and ml/kg) was higher in the ICC group (*p* < 0.001). The number of EPCs in the PRBV content (both ml and ml/kg) were the highest in the ICC group (*p* = 0.002 and *p* = 0.001, respectively). The number of CD34+ HSCs in PRBV content (ml and ml/kg) was similar in all groups, but nonsignificantly higher in the ICC group. The APGAR scores at the first and fifth min were lower in the ICC group (*p* < 0.05). The mean crSO2 values were higher at the 3rd and 10th min in the DCC group (*p* = 0.042 and *p* = 0.045, respectively). cFOE values were higher at the 3rd and 10th min in the ICC group (*p* = 0.011 and *p* < 0.001, respectively).

**Conclusion:**

This study showed that placental transfusion methods, such as DCC and UCM, provide both higher blood volume, more stem cells transfer to the infant, and better cerebral oxygenation in the first minutes of life, whereas many lineages of stem cells is lost to the placenta by ICC with higher residual blood volume. These cord management methods rather than ICC do not require any cost or technology, and may be a preemptive therapeutic source for diseases of the neonatal period.

## Introduction

The fetal–placental blood volume is ~110–115 ml/kg of fetal weight throughout pregnancy. Because the placenta is relatively large in comparison with the fetus at midterm, blood is distributed equally between the fetus and placenta. During a term pregnancy, approximately one-third of the blood flows thorough the placenta and two-thirds flows through the fetus (1). Delayed cord clamping (DCC) reduces residual placental blood to 20% of the feto–placental blood volume in 60 s (s) and to 13% by 35 min (min), but immediate cord clamping (ICC) leaves around 30% of feto–placental blood volume in the placenta (2).

Umbilical cord blood (UCB) has been shown to contain high numbers of hematopoietic stem cells (HSCs) and colony-forming cells, and became an alternative source for transplantation in humans (3, 4). In the following years, many studies and reviews were performed to analyze the maternal and neonatal factors, which may affect the total volume and cell count of collected cord blood units (5–10).

DCC and umbilical cord milking (UCM) are the two methods for transferring residual placental blood to the newborn during the first few minutes of life. Blood volume conservation is critical for infants at birth and during their stay in the neonatal intensive care unit (NICU). This blood contains not only volume, but also 15–20 ml/kg of red blood cells, hundreds of millions to billions of stem cells, and 10–15 ml/kg of plasma. The amount of iron provided by the placental transfusions is enough for a term infant to last 3–8 months (11, 12). The large amount of stem cells represents an autologous transplant, which is important in the development of organ systems, tissue repair, and immunocompetence, as well as lowering the infant's risk of neonatal and age-related diseases (13–15).

Placental transfusion has become the accepted standard in newborn care with shown benefits (16). Placental transfusion provides enhanced vascular perfusion. When compared with keeping the cord intact or unclamped for at least 3 min, ICC decreases hematocrit, blood pressure, blood volume, and iron reserves, increases anemia, and seems to result in less brain myelin and poorer neurodevelopmental skills in term infants (17). The most significant benefits of placental transfusion for preterm infants are decreased mortality rates and better developmental outcomes (18–21).

Based on these findings, we studied the stem cell composition of umbilical cord blood lost by PRBV in healthy term and late preterm infants randomized to receive DCC, UCM, or ICC, and evaluated their delivery room adaptation and cerebral oxygenation.

## Materials and Methods

This prospective, unblinded, and randomized study was conducted at the Ankara University Faculty of Medicine, between October 2019 and October 2020. The study protocol was approved by the local Ethics Committee of the Ankara University Faculty of Medicine under the approval number 07-448-18. A written informed consent form was obtained before delivery from the parents of each infant before randomization. The study was registered at www.clinicaltrials.gov (NCT03983902).

The intervention could not be masked because of the obvious nature of cord clamping. Staff who attended to each birth were asked not to reveal the infant's randomization group in the medical record. Laboratory personnel were masked to the randomization group.

Infants born at or later than 36 gestational weeks from uncomplicated singleton pregnancies were included. Exclusion criteria were parent's refusal to participate, parental desire for umbilical cord blood banking, major congenital anomalies, multiple pregnancies, placental abruption at the time of/or as the indication for delivery, uterine rupture, and severe maternal illness/suspected or proven infection. Prior to delivery, the research staff opened a sequentially numbered opaque randomization envelope. The infants were considered to be randomized to receive DCC, UCM, or ICC at the time the envelope was opened. The demographic findings of included infants were recorded.

### Interventions

DCC was performed by keeping the infant 2–3 cm below the level of the maternal introitus or the incision for 2 min with an intact cord followed by clamping the cord at 2–3 cm from the umbilical stump. Timing of cord clamping was controlled by a stopwatch present in the delivery room.

UCM was performed by grasping the uncut intact umbilical cord and squeezing 25–30 cm of the cord three times at a speed of ~5 cm/s (second) from the placental side to the infant placed at the level of the placenta in cesarean deliveries and 2–3 cm *below* the level of the placenta in vaginal deliveries.

In the ICC group, the infant's umbilical cord was immediately clamped within 20 s after delivery at 2–3 cm from the umbilical stump.

### Measurement of Placental Residual Blood Volume

As soon as the placenta was delivered, the residual volume in the placenta was measured by a simple drainage method by suspending the placenta in a funnel standing at a height of 50 cm. The blood was allowed to drip from the placenta through the cut end of the umbilical cord into a measuring cylinder with gravitation effect. The placental residual blood volume (PRBV) was estimated by measuring the dripped blood into the measuring cylinder, and the PRBV per kg of birth weight was calculated. After draining out the retained blood, the placenta was weighed.

Cord blood samples were collected from placental site from the umbilical vein after cord clamping for complete blood count (1 ml), blood gas analysis (0.5 ml), and flow cytometry (2 ml) with a total amount of 3.5 ml. Cord blood gas analysis and complete blood count were performed from each infant individually. Complete blood counts were immediately analyzed in the laboratory (Coulter LH750 Hematology Analyzer, Beckman-Coulter, Miami, FL, USA) and recorded.

### Determining Umbilical Cord Blood Stem Cells by Flow Cytometry

UCB samples from each group were lysed twice using BD Pharm Lyse lysing buffer (BD Bioscience) at room temperature for 15 min and subsequently washed twice in phosphate-buffered saline with 2% fetal bovine serum to yield total nucleated cells (TNCs).

Staining for EPCs was performed with fluorescence-labeled antibodies for CD45 antigen (KO, Beckman Coulter), CD34 (PE-Cy7, BD Biosciences), CD133 (APC; clone CD133/1, Miltenyl Biotec), and KDR (known as VEGFR2; PE; clone 89106, R&D Systems). As a control, PE-conjugated isotype mouse IgG 1 was used. The absolute number of EPCs and the absolute number of white blood cells were calculated (individually for each sample) per 1 ml of UCB based on the percentage content of these cells as detected by flow cytometry (NAVIOS, Beckman Coulter Corp., Miami, FL, USA). The Kaluza software (Beckman Coulter) was used for analysis.

Staining for HSCs (CD34+) was performed with fluorescence-labeled antibodies for CD45 FITC/CD34 PE (Beckman Coulter) and 7-AAD Viability Dye (Beckman Coulter). As a control, CD45 FITC/IsoClonic PE-conjugated isotype was used. The number of HSCs were calculated (individually for each sample) per 1 ml UCB based on the content of these cells as detected by flow cytometry (NAVIOS, Beckman Coulter Corp., Miami, FL, USA). The Kaluza software (Beckman Coulter) was used for analysis.

### Monitoring of Infants

For the noninvasive monitoring of the heart rate (HR) and arterial oxygen saturation (SpO_2_) a pulse-oximeter sensor (Covidien Nellcor™ Bedside SpO_2_ Patient Monitoring System, MA, USA) was placed on the right wrist of the infant. The near-infrared spectroscopy (NIRS) sensor was placed on the left frontoparietal region. NIRS measurements (crSO_2_) were performed with the INVOS 5100C Cerebral/Somatic Oximeter Monitor (Medtronic, Minneapolis, MN, USA). The mean values for SpO_2_, HR, and crSO_2_ obtained during a 60-s period at minutes 3, 5, and 10 after birth were analyzed. Values of SpO_2_ and HR were stored every second; the sampling rate for crSO_2_ was 8 s. Cerebral fractional oxygen extraction (cFOE) was calculated for each min as (SpO_2_ – crSO_2_)/SpO_2_. As a quality criterion, values of crSO_2_ and SpO_2_ were eliminated when crSO_2_ was higher than SpO_2_.

### Statistical Analysis

Statistical analysis was performed using the Statistical Package for the Social Sciences (version 21). Comparison of categorical data among the three groups of patients was made by the chi-square test. Comparison of continuous data was performed by one-way analysis of variance. *Post-hoc* correction for multiple comparisons was performed with the Tukey's test for unequal samples. The statistical significance was defined as *p* < 0.05. Statistical analysis was performed using the IBM SPSS Statistics version 21.0 for Windows.

## Results

Of the 113 umbilical cord blood samples, 103 were appropriate for analysis (32 DCC, 34 UCM, and 37 ICC). The median times of cord clamping were 120, 15, and 6 s in the DCC, UCM, and ICC groups, respectively.

The demographical features of the groups regarding gestational age, birth weight, gender, and type of delivery were similar. One infant from each group received nasal continuous positive airway pressure; one infant from the UCM group and one infant from the ICC group received positive pressure ventilation (PPV); two, four, and two infants required oxygen at the delivery room from the DCC, UCM, and ICC groups, respectively (*p* = 0.826). None of the infants were admitted to NICU (Table 1).

**Table 1:** The demographic findings of the infants included in the study groups.

	**DCC (*n =* 32)**	**UCM (*n =* 34)**	**ICC (*n =* 37)**	***p*-Value**
Gestational age (w)^*^	38.6 ± 1.1	39 ± 1.2	38.4 ± 1	0.064
Birth weight (g)^*^	3,279 ± 344	3,403 ± 431	3,200 ± 458	0.138
**Gender (male)**, ***n*** **(%)**	18 (56)	19 (56)	14 (38)	0.207
**Type of delivery (CS)**, ***n*** **(%)**	21 (66)	26 (77)	27 (73)	0.608
**APGAR at 1st min** ^**#**^	8 (8–9)	8 (8–9)	8 (7–8)	0.021
**APGAR at 5th min** ^**#**^	10 (9–10)	9 (9–10)	9 (9–10)	0.048
**Management at DR**, ***n*** **(%)**				0.826
***PPV***	0 (0)	1 (3)	1 (3)
***O**_**2**_*	2 (6)	4 (12)	2 (6)
***NCPAP***	1 (3)	1 (3)	1 (3)

* *Data given as mean ± SD*,

# *Median (IQR)*.

*CS, cesarean section; DCC, delayed cord clamping; DR, delivery room; ICC, immediate cord clamping; UCM, umbilical cord milking*.

**Figure 1:**
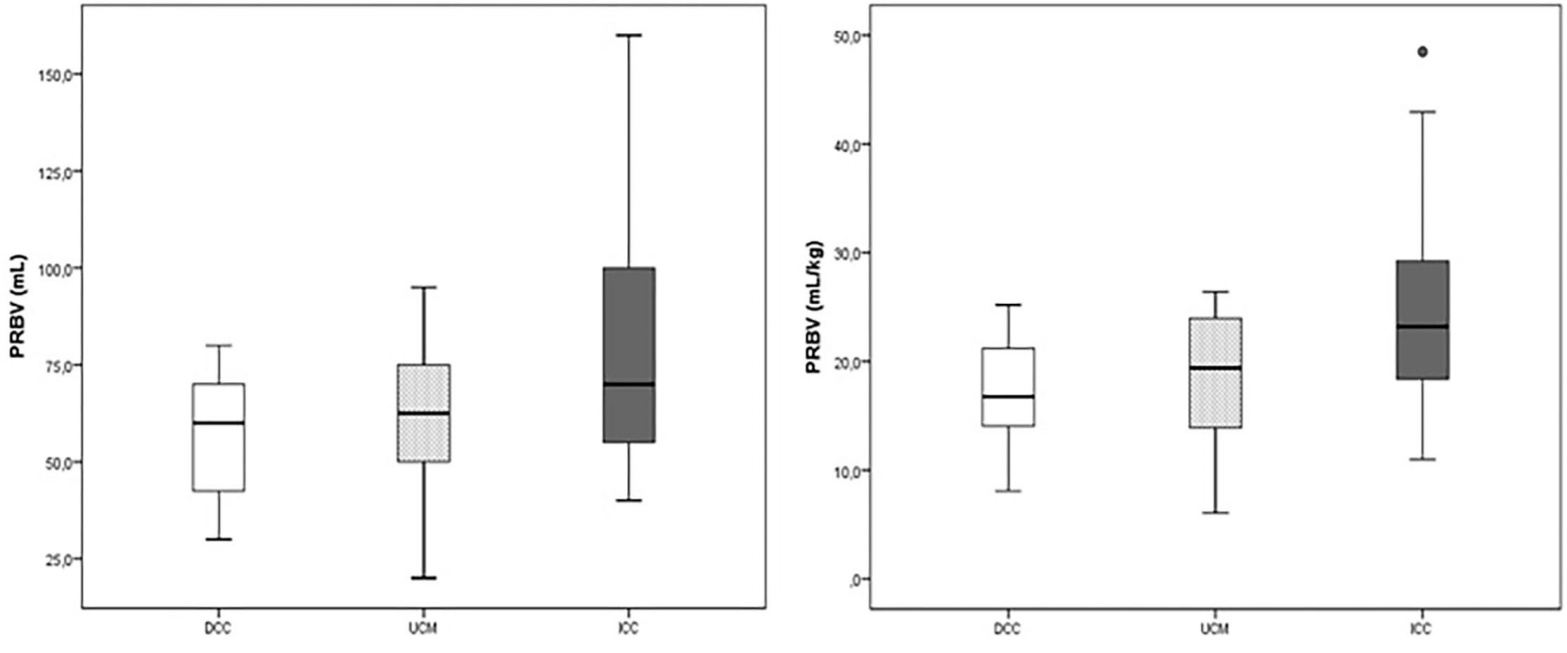
The placental residual blood volume (PRBV) of the delayed cord clamping (DCC), umbilical cord milking (UCM), and immediate cord clamping (ICC) groups (ml and ml/kg).

The placental weight was similar in all groups (*p* > 0.05). The PRBV and PRBV per kg BW were highest in the ICC group (79.5 ± 31.2 ml and 24.8 ± 8.8 ml/kg) (*p* < 0.001), whereas they were similar in the DCC and UCM groups (56.3 ± 15.1 ml and 17.3 ± 4.8 ml/kg vs. 62.6 ± 18.2 ml and 18.6 ± 5.5 ml/kg, *p* = 0.31) (Figure 1).

**Figure 2:**
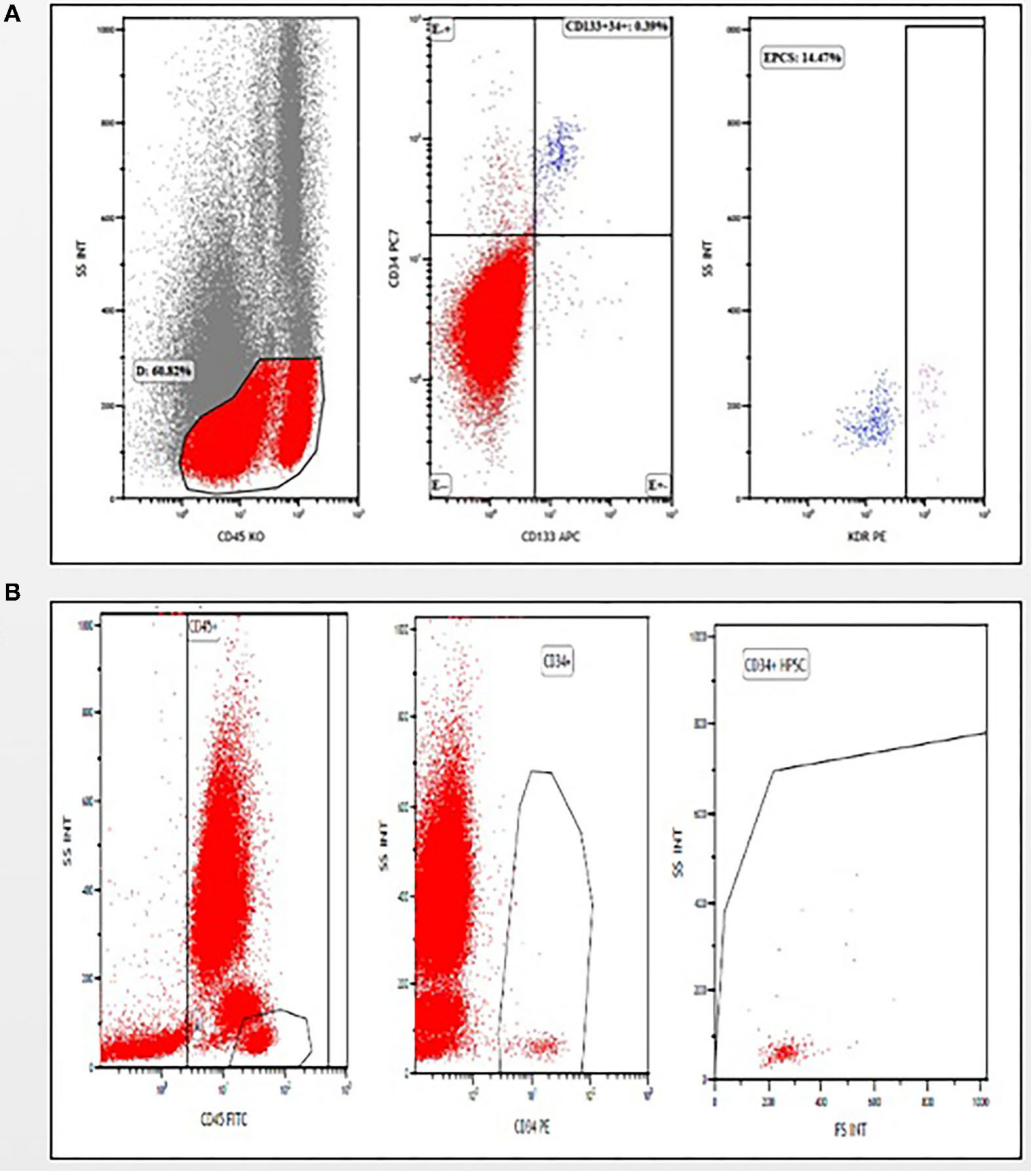
Gating strategies **(A)** for analyzing CD45^−^ cells to identify and enumerate endothelial progenitor cells (EPCs) (CD45^−^/CD133^+^/CD34^+^/KDR^+^), **(B)** for analyzing CD45^+^ cells to identify and enumerate hematopoietic stem cells (HSCs) (CD45^+^/CD34^+^).

We employed multicolor staining by using flow cytometry to analyze the number of EPCs (Figure 2A) and HSCs (Figure 2B) in umbilical cord blood.

The percentage of EPCs and the number of CD34+ HSCs per μl in UCB were similar in all groups (*p* = 0.065 and 0.961, respectively). The number of EPCs were higher in the PRBV (ml and ml/kg) of the ICC group (*p* = 0.002 and *p* = 0.001, respectively), whereas it was similar in the DCC and UCM groups (*p* > 0.05). The number of CD34+ HSCs was not different in the PRBVs (ml and ml/kg) of the three groups (*p* = 0.123 and 0.075, respectively) (Table 2).

**Table 2:** The numbers of endothelial progenitor cells (EPCs) and CD34+ hematopoietic stem cells (HSCs) following cord management.

	**DCC (*n =* 32)**	**UCM (*n =* 34)**	**ICC (*n =* 37)**	***p*-Value**
**EPC (cells/ml)**	2.6 × 10^3^ ± 2.3 × 10^3^	2.8 × 10^3^ ± 1.7 × 10^3^	3.9 × 10^3^ ± 3.1 × 10^3^	0.065
**PRBV EPC (cells)**	15.1 × 10^4^ ± 14.1 × 10^4^	16.3 × 10^4^ ± 96 × 10^4^	32.2 × 10^4^ ± 33.8 × 10^4^	0.002
**PRBV EPC (cells/kg)**	4.6 × 10^4^ ± 4.5 × 10^4^	4.7 × 10^4^ ± 2.7 × 10^4^	10.1 × 10^4^ ± 10.3 × 10^4^	0.001
**CD34+** **HSC (cells/ml)**	35.6 × 10^3^ ± 21.8 × 10^3^	34.9 × 10^3^ ± 18 × 10^3^	34.2 × 10^3^ ± 21 × 10^3^	0.961
**PRBV CD34+** **HSC (cells)**	2.1 × 10^6^ ± 1.5 × 10^6^	2.2 × 10^6^ ± 1.3 × 10^6^	2.9 × 10^6^ ± 2.5 × 10^6^	0.123
**PRBV CD34+** **HSC (cells/kg)**	0.64 × 10^6^ ± 0.46 × 10^6^	0.64 × 10^6^ ± 0.38 × 10^6^	0.89 × 10^6^ ± 0.72 × 10^6^	0.075

*All data given as mean ± SD*.

*DCC, delayed cord clamping; EPC, endothelial progenitor cell; HSC, hematopoietic stem cell; ICC, immediate cord clamping; PRBV, placental residual blood volume; UCM, umbilical cord milking*.

**Figure 3:**
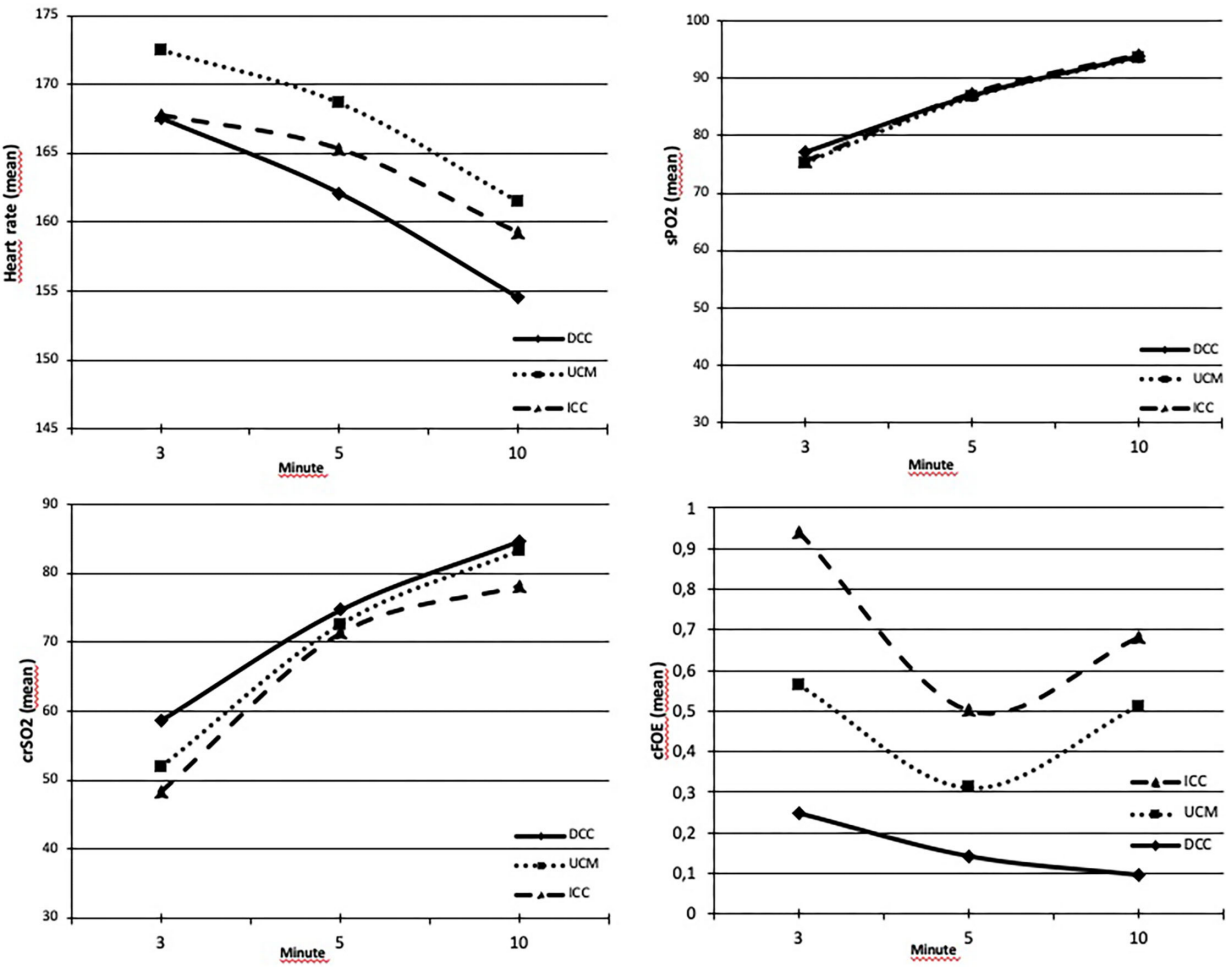
**(A)** Heart rates (HRs) were similar between groups at the 3rd, 5th, and 10th min (*p* > 0.05). **(B)** SpO2 values were similar between groups at the 3rd, 5th, and 10th min (*p* > 0.05). **(C)** crSO_2_ values were higher at the 3rd and 10th min in the DCC group (*p* = 0.042 and *p* = 0.045, respectively) and similar in all groups within normal range at the 5th min (*p* = 0.61). **(D)** cFOE values were the highest at the 3rd and 10th min in the ICC group (*p* = 0.011 and *p* < 0.001, respectively).

The APGAR scores at the first and fifth min were lower in the ICC group (*p* = 0.012 and *p* = 0.048). There were no differences in terms of HR and SpO2 at the 3rd, 5th, and 10th min, and crSO_2_ and cFOE values at the 5th min between the groups (*p* > 0.05). The mean crSO_2_ values were higher at the 3rd and 10th min in the DCC group (*p* = 0.042 and *p* = 0.045, respectively). These values were similar between the UCM and ICC groups at this timepoints (*p* = 0.39 and *p* = 0.09, respectively). cFOE values were higher in the ICC group at both the 3rd and 10th min (*p* = 0.011, and *p* < 0.001, respectively), whereas these values were similar between the DCC and UCM groups at the 3rd min (*p* = 0.08), and higher in the UCM group than in the DCC group at the 10th min (*p* < 0.001) (Figure 3).

The blood gas analysis and hematological parameters were comparable in the three groups (*p* > 0.05) (Table 3).

**Table 3:** Blood gas values and hematological parameters.

	**DCC (*n =* 32)**	**UCM (*n =* 34)**	**ICC (*n =* 37)**	***p*-Value**
**pH** ^*^	7.32 ± 0.08	7.34 ± 0.05	7.30 ± 0.09	0.074
${\text{HCO}}_{3}^{*}$	20.02 ± 2.7	20.31 ± 1.9	20.04 ± 2.6	0.857
BE^*^	−4.6 ± 3.6	−4.0 ± 2.7	−4.8 ± 2.8	0.517
Lactate^*^	2.1 ± 0.87	1.8 ± 0.95	2 ± 1.33	0.610
Hemoglobin (g/dl)^*^	15.3 ± 1.7	15.7 ± 1.2	15.9 ± 1.8	0.508
Hematocrit (%)^*^	45.2 ± 5.7	46.8 ± 4.2	48.2 ± 5.3	0.213
**Leucocyte (mm** ^**3**^ )^*^	12,972 ± 3,138	13,214 ± 3,485	13,402 ± 3,627	0.961
**Thrombocyte (mm** ^**3**^ )^*^	255,500 ± 78,258	253,706 ± 76,592	240,875 ± 85,626	0.684

* *Data given as mean ± SD*.

*BE, base excess*.

## Discussion

This study characterizes the differences in stem cell composition of PRBV and parameters in transition period after birth based on the umbilical cord management strategies. It has been shown that DCC and UCM provide higher blood volume and stem cell transfer to infants, and DCC provides better cerebral oxygenation. In addition to these findings, we have demonstrated that ICC resulted in losses of more EPCs and CD34+ HSCs through higher placental residual blood volume, and was associated with lower APGAR scores and lower cerebral oxygenation in subsequent minutes after delivery.

Keeping the umbilical cord intact after birth helps the baby's circulation transition from the fetal to neonatal period and allows for considerable blood flow through the placenta. PRBV measurements can be used to estimate the amount of blood transfused to the infant in the initial few minutes after birth because there is currently no ethical means to assess the transfused blood volume in healthy term infants. With a DCC of 5 min, Mercer et al. showed that term infants have lower amounts of PRBV with higher hemoglobin and hematocrit in the first 2 days of life without an increase in jaundice, polycythemia, or other adverse effects (22). The same results (after DCC of 3 min) with larger groups were shown by Andersson et al. (23). A DCC of at least 60 and 180 s in healthy term infants reduced the rate of anemia and iron deficiency, improved the hemodynamic parameters (i.e., peripheral arterial oxygen saturation, HR, cardiac output, and cerebral oxygenation) with no increase in the rates of phototherapy in both high- and limited-resource environments (24). We have also demonstrated lower PRBVs with a DCC of 2 min and UCM compared with ICC with similar placental weights and hematological parameters in term and late preterm infants.

UCB contains a high concentration of stem cells, such as HSCs, endothelial cell precursors, mesenchymal progenitors, and pluripotent/multipotent stem cells (25–27). After the first successful cord blood transplantation (4), UCB was accepted to be an available source for transplantation, which was previously considered as medical waste. Over the years, the volume and cell content of cord blood were evaluated in studies, which were mostly designed for cord blood banking. The influence of maternal and neonatal factors, such as gestational age, birth weight, type of delivery, time of clamping the cord, etc., on the volume and cell content of collected cord blood were investigated (9). It has been demonstrated that early clamping resulted in higher mean volume and TNCs in cord blood units compared with DCC (28). Further studies are needed about the effects of varying durations of cord clamping on the collection volume and the cell content, which is not the objective of this study.

The placental and umbilical cord contracting with blood pumping toward the newborn during birth results in the first natural stem cell transplant. The newborn is deprived of both the volume and stem cells that he/she could have received if the umbilical cord was clamped and cut immediately. This intentional loss of stem cells at birth is thought to predispose infants to diseases like chronic lung disease, asthma, diabetes, cerebral palsy, infection, and cancer. Furthermore, it is argued that if the birth process is traumatic for the infants, it is possible for stem cells to help in recovery (14, 29, 30). Our results revealed that the placental reserve volume exists to provide placental to fetal transfusion with DCC or UCM as evidenced by lower EPCs and CD34+ HSCs in PRBV. Infants who were randomized to ICC left more residual blood volume behind in their placentas with more stem cells wasted. The therapeutic dose of various commercially available stem cells is still unknown, but a dose of 1 × 10^6^ has been used in very few clinical trials on BPD and HIE in newborns (31–33). The difference in the numbers of EPCs and CD34+ HSCs wasted by ICC compared with DCC and UCM approximately seemed to be ¼ to half of this therapeutic dose in this study.

Continuous monitoring of regional tissue oxygenation with NIRS provides indirect information on brain perfusion. Most of the studies measured the changes in cerebral oxygenation during the transition period after delivery in preterm infants. Placental transfusion optimizes tissue perfusion and should also have an effect on cerebral perfusion, potentially lowering the risk of hypoxic ischemic brain injury in these newborns (34, 35). Baenziger et al. showed that DCC improved cerebral oxygenation in preterm infants in the first 24 h (36). In the study of Finn et al. comparing the effects of umbilical cord clamping methods (immediate clamping, milking, and delayed clamping) on cerebral activity and oxygenation at 6–12 h after birth in babies born before 32 weeks of gestation, it was reported that similar results were obtained with these three methods (37). A DCC of 5 min resulted in higher mean arterial blood pressure and cerebral tissue oxygenation than a DCC of 1 min in the first 12 h after birth in term infants at risk for resuscitation (38). Similarly, DCC seems to be beneficial regarding hypovolemia and hypotension, both of which can affect dynamics of cerebral blood flow (39). However, only a few studies measured the cerebral oxygenation during the transition period in healthy term infants (40–42), and there are very limited data on how the duration of cord clamping affects cerebral hemodynamics in term infants. This study demonstrated no differences in terms of HR and SpO2 at the 3rd, 5th, and 10th min, and cerebral oxygenation values at the 5th min between the groups. On the other hand, crSO_2_ values were the highest at the 3rd and 10th min after birth in infants who received DCC, but similar in the UCM and ICC groups. The cFOE values were higher in infants in the ICC group at the 3rd and 10th min after birth, which were similar between the DCC and UCM groups at the 3rd min and higher in the UCM group than in the DCC group at the 10th min after birth. These results suggested that DCC and UCM allow infants to increase the pulmonary blood flow before placental circulation is lost and initiates improved cerebral hemodynamic stability in the very early minutes of life. The beneficial effect of DCC on cerebral oxygenation at the 3rd and 10th min has not been detected at the 5th min of life, but both crSO_2_ and cFOE values were in normal range in all the groups at that timepoint. Although the incidence of need for resuscitation at the delivery room were similar between the groups, more infants from the UCM group received PPV or O_2_ at the delivery room, which may have caused not to be able to detect the positive effect of UCM statistically.

Cord blood acid–base parameters measured at birth have been found to be different due to clamping time and to be altered in DCC compared with ICC (43, 44). DCC up to 120 s has either no effect or only a slight influence on cord blood acid–base balance with no clinically significant effect, according to a recent systematic review documenting umbilical blood gas measurements of vaginally delivered healthy term singletons (45). Cord blood gas values obtained after DCC or UCM or ICC were not different in our study, which consisted of healthy infants.

To our knowledge, this is the first randomized study that evaluated PRBV and the stem cell composition with the three types of umbilical cord management strategies as DCC, UCM, and ICC, and also evaluates the hematological parameters at birth and cerebral oxygenation values at the first minutes after birth. The current study has some limitations. To eliminate potential confounders, only late preterm and term infants were enrolled in this study. The groups were demographically similar, and the delivery mode and sex were evenly distributed. To avoid obtaining blood samples from healthy infants, which we assumed would not reflect the composition of the cord blood, we did not analyze the stem cell composition of peripheral blood obtained directly from the infants after clamping of the cord or later.

## Conclusion

In this study, similar results were obtained with both DCC and UCM in terms of both volume and stem cell composition and better cerebral oxygenation at the first minutes of life with DCC and UCM with respect to ICC. DCC and UCM are easy to perform interventions with no cost in all environments. Placental transfusion methods are known to improve hemodynamics and organ perfusion, and may also provide stem cells that can be used for preemptive therapeutic purposes in neonatal diseases.

## Data Availability Statement

The raw data supporting the conclusions of this article will be made available by the authors, without undue reservation.

## Ethics Statement

The studies involving human participants were reviewed and approved by Ankara University Faculty of Medicine Local Ethics Committee (Approval No. 07-448-18). Written informed consent to participate in this study was provided by the participants' legal guardian/next of kin.

## Author Contributions

EO, SH, DG, and SA gave substantial contribution to the article conception and design. EO, SH, DG, EK, OE, BA, AK, and FS participated in the acquisition of data. EO and SH drafted the manuscript. FD, AI, and SA critically revised the manuscript. All authors gave their final approval to this manuscript and agree to be accountable for all aspects of the work ensuring integrity and accuracy.

## Funding

This project has been funded within the scope of the TUBITAK-1002 with project number 218S439.

## Conflict of Interest

The authors declare that the research was conducted in the absence of any commercial or financial relationships that could be construed as a potential conflict of interest. The handling editor AE declared past co-authorships with one of the authors EO and the absence of any ongoing collaboration with any of the authors.

## Publisher's Note

All claims expressed in this article are solely those of the authors and do not necessarily represent those of their affiliated organizations, or those of the publisher, the editors and the reviewers. Any product that may be evaluated in this article, or claim that may be made by its manufacturer, is not guaranteed or endorsed by the publisher.
